# miR-485-5p alleviates Alzheimer’s disease progression by targeting PACS1

**DOI:** 10.1515/tnsci-2020-0177

**Published:** 2021-09-14

**Authors:** Chuan He, Caixia Su, Wentong Zhang, Qi Wan

**Affiliations:** Department of Rehabilitation Medicine, Jiangsu-Shengze Hospital affiliated to Nanjing Medical University, Suzhou 215228, Jiangsu, China; Department of Neurological Medicine, The First Affiliated Hospital of Nanjing Medical University, 300 Guangzhou Road, Nanjing 21000, Jiangsu, China

**Keywords:** Alzheimer’s disease, pericytes, miR-485-5p, PACS1

## Abstract

Alzheimer’s disease (AD) is a common dementia and a heterogeneous disease. Previous research has validated that microRNAs (miRNAs) are pivotal regulators in the initiation and development of tremendous diseases including AD. MicroRNA-485-5p (miR-485-5p) was reported to be an important participant implicated in several neurological diseases, but its role in AD still needs to be further investigated. In this research, we explored the biological function of miR-485-5p in AD. RT-qPCR revealed that miR-485-5p expression was downregulated in the hippocampus of APP/PS1 mice. Additionally, miR-485-5p overexpression facilitated the learning and memory capabilities of APP/PS1 mice according to Morris water maze test, fear conditioning test, and immunofluorescent staining. Moreover, CCK-8 assay, flow cytometric analysis, and western blot analysis suggested that miR-485-5p overexpression promoted pericyte viability and prohibited pericyte apoptosis in APP/PS1 mice. Mechanistically, miR-485-5p directly targeted PACS1 in pericytes, as shown in a luciferase reporter assay. In rescue assays, PACS1 overexpression countervailed the effect of miR-485-5p overexpression on pericyte viability and apoptosis. In conclusion, miR-485-5p ameliorates AD progression by targeting PACS1.

## 1 Introduction

Alzheimer’s disease (AD) is a neurodegenerative brain disease among the elderly population [1], which can be caused by multiple factors including genetic, biological, and psychosocial factors [2]. The pathogenesis of AD is characterized by neuronal loss and atrophy as well as neurodegeneration such as the degeneration of pericytes [3,4]. Pericytes are positioned in the neurovascular unit to maintain functions of central nervous system (CNS) by cooperating with neighboring cells via complicated signaling pathways [5,6]. Previous research demonstrated that the damage of pericytes may lead to blood-brain barrier (BBB) breakdown, one of the early signs of neurodegenerative diseases including AD [7,8]. Moreover, the progressive degeneration of pericytes can boost Alzheimer-like neurodegeneration in transgenic APP/PS1 mice according to previous studies, highlighting that prohibiting pericyte apoptosis might be beneficial to prevent or slow down the development of AD [9,10]. Hence, we established a murine AD model to investigate the learning and memory abilities of APP/PS1 mice and the viability and apoptosis of pericyte in this study.

During the past decades, microRNA (miRNA), a member of noncoding RNA family, has become a major class of regulatory molecules implicated in neurological diseases and disorders including AD [11]. miRNA has approximately 20 nucleotides in length and plays a crucial role in regulating gene expression at the posttranscriptional stage [12]. Previously, many miRNAs were reported to be associated with the pathogenesis and progression of AD [13]. For example, miR-455-3p is reported to be a peripheral biomarker and a potential therapeutic target for AD [14]. The cognitive decline in AD may be prevented or slowed by reversing the abnormally expressed miR-30b in the brain [15]. MicroRNA-485-5p (miR-485-5p) was reported to suppress the development of AD by repressing the expression of beta-secretase-1 (BACE1) [16]. However, research on the functions of miR-485-5p in AD is quite limited, and the potential molecular mechanism of miR-485-5p in AD pathology still needs further exploration. Thus, we explored the function and mechanism of miR-485-5p and its target gene PACS1 in the progression of AD.

Phosphofurin acidic cluster sorting protein 1 (PACS1) is a widely studied mRNA that participates in the regulation of versatile biological processes [17–20]. Importantly, PACS1 is verified to prevent the breakdown of amyloid precursor protein (APP) into Aβ peptides [21], a hallmark of AD [22]. However, the specific function and potential mechanism of PACS1 in AD remain uncharacterized. Herein, we investigated the role of PACS1 in AD since PACS1 was predicted to be a target gene of miR-485-5p based on bioinformatics analysis.

In conclusion, the aim of the study is to further disclose the function and mechanism of miR-485-5p in AD progression. The study might provide novel insight into the investigation of molecules in AD.

## 2 Materials and methods

### 2.1 Animals

Male APP/PS1 transgenic mice (*n* = 30) and wild-type C57BL/6 mice (WT; *n* = 10) were purchased from the Institute of Laboratory Animal Science, Chinese Academy of Medical Sciences. All mice were kept in standard housing condition and given sufficient food and water.

**Ethical approval:** The study related to animal use has been complied with all the relevant national regulations and institutional policies for the care and use of animals. The animal handling procedures were approved by the Institutional Animal Care and Use Committee of The First Affiliated Hospital of Nanjing Medical University (Jiangsu, China).

### 2.2 Animal grouping and treatment

To explore the role of miR-485-5p in APP/PS1 mice, miR-485-5p expression in the hippocampus of APP/PS1 mice was first examined. Hippocampus was collected from APP/PS1 mice (*n* = 10) and WT mice (*n* = 10). RT-qPCR was conducted to examine miR-485-5p expression in the hippocampus of these mice. Additionally, PACS1 expression in APP/PS1 mice’s hippocampus was also examined, with PACS1 expression in WT mice’s hippocampus as a control.

Twenty APP/PS1 mice were randomly selected and divided into two groups: negative control (NC) group (*n* = 10, mice were injected with empty lentiviral vector) and miR-485-5p group (*n* = 10, APP/PS1 mice were treated with the injection of lentiviral vector containing miR-485-5p fragments). The lentiviral vector (pEZX-MR03) containing the fragments of miR-485-5p (miR-485-5p) and empty lentiviral vector (NC) were synthesized by Genecopoeia (Rockville, MD, USA). Lentiviral particles were generated as previously described [23]. After being anesthetized by pentobarbital sodium, the mice were immobilized by a stereotaxic apparatus. Afterwards, the hippocampus of mice was injected with 2 μL lentiviral vector containing the fragments of miR-485-5p or empty lentiviral vector using a 27-gauge needle and a Hamilton 5 μL syringe at 0.4 μL/min for 5 min. One week later, mice were used for behavioral tests and then sacrificed to collect their brains. The bloods were collected before LV injection.

### 2.3 Cells and cell treatment

The murine brain pericytes were separated from the micro vessel fragments of mouse cortex and hippocampus as previously described [24]. The separated micro vessels were incubated in Pericyte Medium (ScienCell, USA) containing fetal bovine serum (10 mL), basal medium (500 mL), penicillin/streptomycin solution (5 mL), and pericyte growth supplement (5 mL). After 48 h, the micro vessels that were not adherent were cleared. For cell treatment, pericytes were cultured with or without Aβ40 for consecutive 3 days. Medium with 0, 2.5, 5, and 10 mM of Aβ40 was replaced every 2 days.

### 2.4 Cell transfection

The level of PACS1 was overexpressed by pcDNA3.1/PACS1 vector with empty pcDNA3.1 as a control. miR-485-5p mimics were employed to upregulate miR-485-5p expression and NC mimics was set as a corresponding negative control. All plasmids (GenePharma, Shanghai, China) were transfected into pericytes by Lipofectamine 2000 (Invitrogen, USA). After 48 h of transfection, the efficiency was examined by RT-qPCR or western blot.

### 2.5 RNA extraction and real-time quantitative polymerase chain reaction (RT-qPCR)

After being anesthetized using pentobarbital sodium, brain tissues were collected. Next, the brain tissues of the mice were homogenized by TRIzol reagent (Invitrogen, USA) on ice to extract total RNA. Afterwards, the extracted RNA was reverse transcribed into cDNA utilizing High Capacity cDNA Reverse Transcription Kits (Applied Biosystems, Foster City, CA, USA). RT-qPCR analysis was conducted employing a SYBR^®^ Premix Ex TaqTM II reagent kit (RR820A, Takara) on a 7500 Real-Time PCR System (Applied Biosystems). U6 served as the endogenous control for miR-485-5p. The detection results for mRNAs (PACS1, MON2, TCTA, SOX10, and PPARGC1A) were normalized to GAPDH. Expression fold changes were calculated adopting the 2^−ΔΔCt^ method. The raw data of the 2^−ΔΔCt^ can be found in Table S1.

### 2.6 Morris water maze test

The spatial memory of the mice was assessed using Morris water maze test according to the previous study [25]. The platform (10 cm in diameter) was placed 1 cm below the surface of water. Each mouse was given 1 min to arrive at the platform. If it failed, it would be led to the platform and kept there for 15 s. At the training stage, the mice needed to complete four trials a day. There should be at least 20 min between each trial. For each trial, the mice were put into the water at the randomly selected quadrant. The elapsed time for searching for the platform was taken as the escape latency. The training lasted for 5 days. At the testing stage, we took off the platform from water and the mice were given 1 min to search for the platform. The duration in the target quadrant was recorded.

### 2.7 Measurement of Aβ42 and Aβ40

Enzyme-linked immunosorbent assay (ELISA) was carried out to quantify the concentration of Aβ42 and Aβ40 in the hippocampus of APP/PS1 mice according to the previous study [25]. Briefly, after anesthesia, the brain tissues of the mice were obtained and homogenized in the RIPA buffer (89900, Thermo Fisher Scientific) on ice. Next, protein samples were obtained after the mice brain tissues were centrifuged at 12,000 rpm for 10 min. The BCA assay was adopted to measure the concentration of protein, which was then transferred to 96-well plate. The concentration of Aβ42 and Aβ40 was quantified by the Amyloid beta 40 Mouse ELISA Kit (Cat #KMB3481, Invitrogen) and the Amyloid beta 42 Mouse ELISA Kit (Cat # KMB3441, Invitrogen), respectively. Results are presented as ng Aβ42 and Aβ40 per mg total protein.

### 2.8 Fear conditioning test

The test was employed to evaluate learning and memory abilities of mice based on a previous study [22]. The mice were put in a chamber box with grid floor. The chamber was cleaned using 90% ethanol after each trial. On the first day, the mice were kept at the chamber for 3 min, then they were given two footshocks (0.5 mA; 2 s each time) with a 1–4 min interval. Afterwards, the mice were placed in the same chamber for 1 min and then put back to the cage. After 24 h, the mice were put in the same chamber for 3 min with no footshock for fear memory test. Total freezing time was recorded and evaluated.

### 2.9 Western blot analysis

Tissues and cells were lysed by the RIPA lysis buffer. Protein samples were isolated by SDS-PAGE and then moved to PVDF membranes. Next, the samples were covered with 5% nonfat milk powder for 1 h. Primary antibodies (Abcam, Cambridge, UK) against PACS1 (ab208171, 1:1,000), Bax (ab32503, 1:1,000), Bcl-2 (ab196495, 1:500), cleaved caspase-3 (ab214430, 1:5,000), and GAPDH (ab8245, 1:2,000) were incubated with the membranes overnight at 4°C. After secondary antibodies were incubated with the membranes at 37°C for 1 h, an ECL Plus reagent (Applygen Technologies Inc., Beijing, China) was employed to develop the bands. ImageJ software (National Institutes of Health, Bethesda, MA, USA) was utilized to quantify the result of western blot. GAPDH was set as a loading control. To assess cell apoptosis, apoptosis ratio was measured by the ratio of Bcl-2/Bax protein level.

### 2.10 Luciferase reporter assay

The wild-type (Wt) or mutant (Mut) sequence of PACS1 3′-untranslated region (3′-UTR) was inserted into pmiRGLO reporters (Promega, Madison, WI, USA). PACS1-Wt or PACS1-Mut was co-transfected, respectively, with miR-485-5p mimics or NC mimics into pericytes. Lipofectamine 2000 was applied to perform the transfection. The luciferase assay was carried out employing a dual-luciferase reporter assay system kit (Promega) after 48 h of transfection. The luciferase activity was evaluated by the Modulus single-tube multimode reader (Promega).

### 2.11 Immunofluorescent staining

Brain slices were coated by 5% normal goat serum for 1 h at room temperature. Then, these slices were incubated with primary antibody of anti-CD13 (ab108310, Abcam; 1:200) overnight. Next, after being washed with phosphate buffer solution (PBS), brain slices were incubated with the secondary antibody. Subsequently, Dylight 488-conjugated tomato lectin (1:100, Vector Laboratories) was applied to stain the sections. Fluorescent mounting medium (Dako, Carpinteria, CA, USA) was utilized to cover the sections and a microscopy was used to capture brain micro vessel. Additionally, brain sections were stained with 0.2% thioflavin-S (T1892, Sigma-Aldrich). After being repeatedly washed with PBS, brain sections were observed by an IX53 fluorescence.

### 2.12 Cell counting kit-8 (CCK-8) assay

The assay was carried out to detect the viability of pericytes. In brief, pericytes (Aβ40) were plated to 96-well plates overnight. After 48 h, the CCK-8 reagent was supplemented to the cells for another 2 h of incubation at 37°C. Finally, a multifunctional microplate reader (SpectraMax M5, Sunnyvale, CA, USA) was used to assess the absorbance of the samples at 450 nm.

### 2.13 Flow cytometric analysis

Pericyte apoptosis was evaluated adopting the Annexin V-FITC/PI Kit (Life Technologies, Carlsbad, CA, USA). In brief, cells were washed with PBS and cultured with Annexin V-FITC/PI Kit after being exposed to Aβ40 for 24 h. The cell suspension was subjected to flow cytometric analysis using a flow cytometer.

### 2.14 Statistical analysis

Data analysis was performed with SPSS 23.0 (IBM SPSS, Chicago, IL, USA). All data are expressed as the mean ± standard deviation (SD). Statistical significance among more than 2 groups was calculated using one-way ANOVA followed by Tukey’s *post hoc* test. Difference between 2 groups was analyzed with Student’s *t* test. The value of *p* < 0.05 was regarded to be statistically significant.

### 2.15 Bioinformatics analysis

The target genes of miR-485-5p were predicted from miRDB (http://miRdb.org/cgi-bin/search.cgi) [26]. A total of 574 mRNAs were found to share binding site with miR-485-5p (Table S1). The top five mRNAs (PACS1, MON2, TCTA, SOX10, and PPARGC1A) were identified for the study.

## 3 Results

### 3.1 miR-485-5p ameliorates learning and memory abilities of APP/PS1 mice

**Figure 1:**
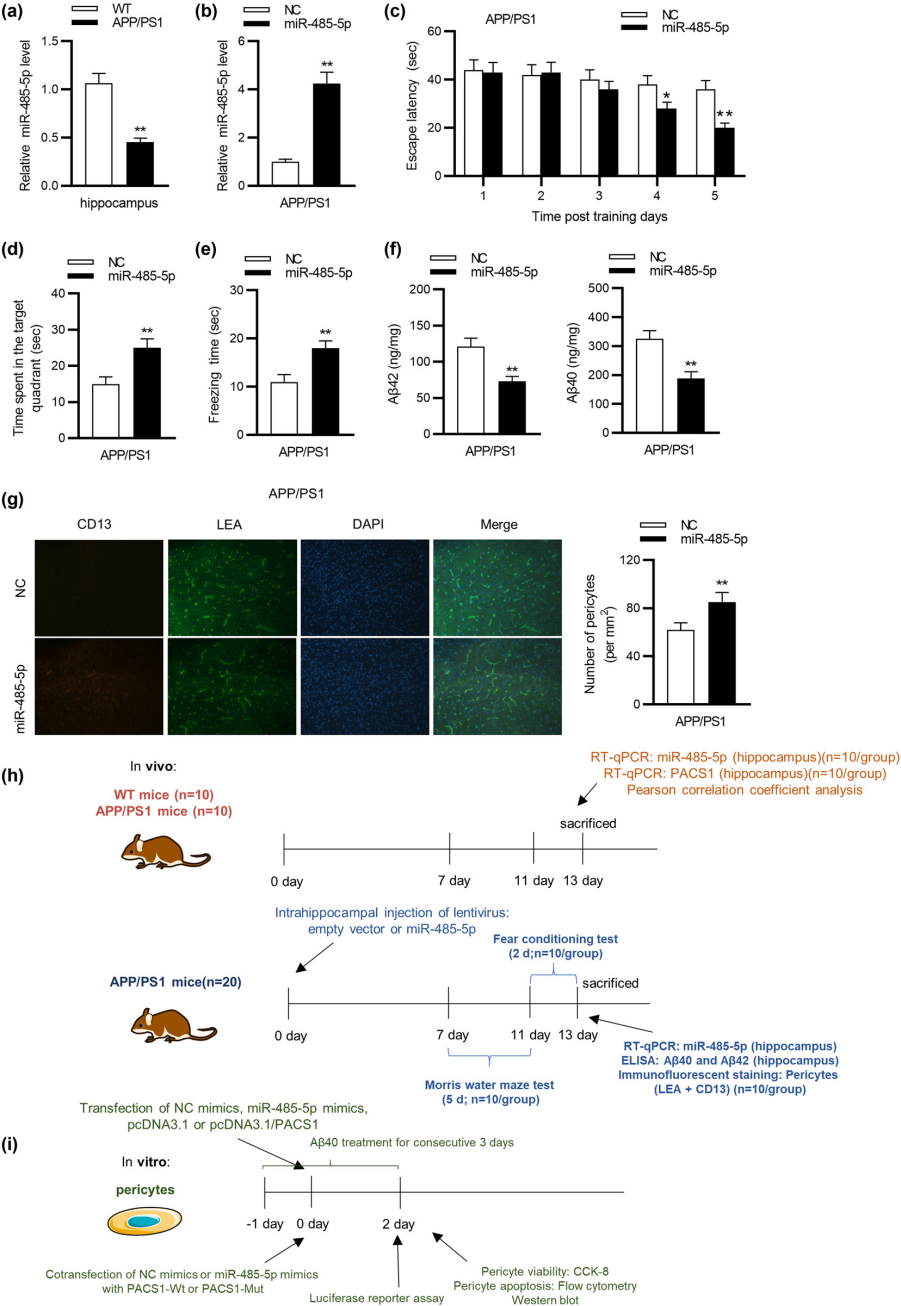
Overexpression of miR-485-5p alleviates cognitive deficits of APP/PS1 mice. (a) Nine-month-old APP/PS1 mice (*n* = 20; 10/group) were treated with injection of lentiviral empty vector or lentiviral miR-485-5p expressing vector into the hippocampus. miR-485-5p level in the hippocampus of APP/PS1 mice was detected using RT-qPCR analysis. (b) RT-qPCR analysis was conducted to measure the transfection efficiency of miR-485-5p overexpression in APP/PS1 mice (*n* = 20; 10/group). (c) Spatial learning of APP/PS1 mice was detected as escape latency (s) in water maze (*n* = 20; 10/group). (d) The time spent in the target quadrant for APP/PS1 mice was recorded (*n* = 20; 10/group). (e) For the fear memory test, the total freezing time was studied on test day (*n* = 20; 10/group). (f) The levels of Aβ42 and Aβ40 in brain tissues of WT and APP/PS1 mice (*n* = 20; 10/group) were examined by ELISA assay. (g) The representative immunofluorescent images of CD13-positive pericytes (red) and lectin-positive capillary endothelium (green) in APP/PS1 mice with or without miR-485-5p overexpression were exhibited. Quantification of CD13-positive pericytes in APP/PS1 mice (*n* = 20; 10/group) with or without miR-485-5p overexpression was displayed. (h) The experimental schedule of *in vivo* assays was shown in the diagram. (i) The *in vitro* assays were conducted according to the diagram. ^*^ *p* < 0.05, ^**^ *p* < 0.01.

RT-qPCR analysis showed that miR-485-5p expression was significantly (student’s *t* test; *p* < 0.01) downregulated in the hippocampus of APP/PS1 mice compared with its expression in that of WT mice (Figure 1a). After the injection of LV-miR-485-5p into APP/PS1 mice, hippocampal miR-485-5p level was prominently (student’s *t* test; *p* < 0.01) overexpressed, as suggested by RT-qPCR (Figure 1b). According to the Morris water maze test, miR-485-5p overexpression obviously (student’s *t* test; *p* < 0.05) shortened the escape latency of APP/PS1 mice compared with that of mice in the NC group on day 4 and day 5 (Figure 1c). In addition, miR-485-5p overexpression remarkably (student’s *t* test; *p* < 0.01) prolonged the time that APP/PS1 mice spent in the target quadrant (Figure 1d). Subsequently, the results of fear conditioning test demonstrated that the mice with enhanced miR-485-5p expression showed distinctly (student’s *t* test; *p* < 0.01) longer freezing time compared with those mice in the NC group (Figure 1e). In addition, the levels of Aβ42 and Aβ40 were remarkably (student’s *t* test; *p* < 0.01) decreased in miR-485-5p group compared with that in the NC group (Figure 1f). Furthermore, immunofluorescent staining was conducted to confirm the effect of miR-485-5p on pericytes of APP/PS1 mice. The results reflected that overexpressed miR-485-5p significantly (student’s *t* test; *p* < 0.01) augmented the number of pericytes in the hippocampus of APP/PS1 mice (Figure 1g). The experimental schedule of both *in vivo* and *in vitro* assays was shown in Figure 1g and h.

### 3.2 miR-485-5p represses Aβ40-induced pericyte apoptosis

**Figure 2:**
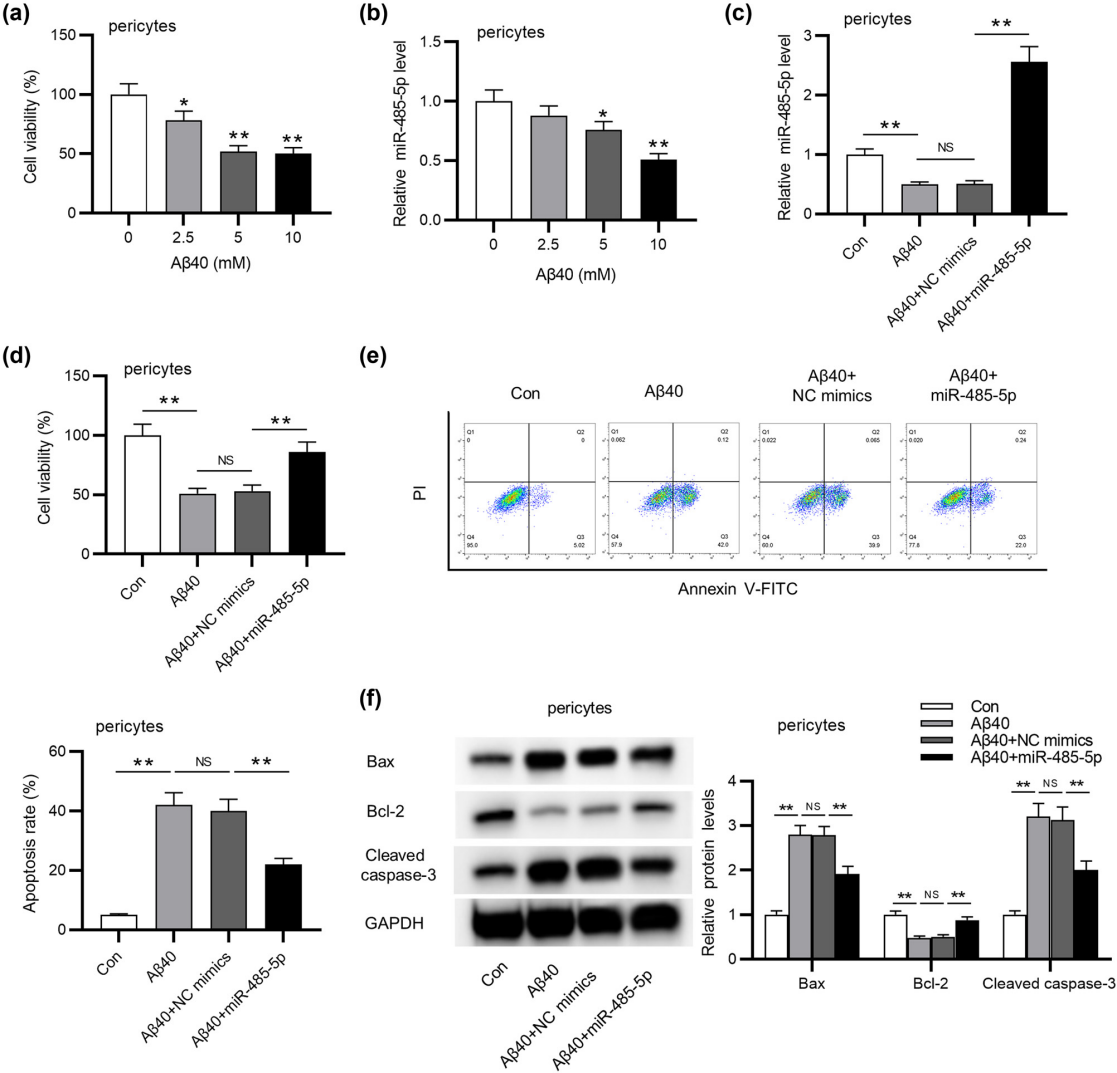
miR-485-5p improves pericyte viability and suppresses pericyte apoptosis of APP/PS1 mice. (a) The effect of 0, 2.5, 5, 10 mM Aβ40 on the viability of pericyte was detected using CCK-8 assay. (b) RT-qPCR analysis was performed to measure miR-485-5p level in pericyte treated with the above concentration of Aβ40. (c) RT-qPCR analysis was conducted to measure miR-485-5p level in pericytes transfected with Aβ40, Aβ40 + NC mimics, or Aβ40 + miR-485-5p. (d) The viability of pericytes in the above four groups was determined by CCK-8. (e) The apoptosis of pericytes in the above groups was determined by flow cytometry. (f) Western blot analysis was conducted to evaluate the levels of proteins (Bax, Bcl-2, and cleaved caspase-3) associated with cell apoptosis. The apoptosis ratio was measured by the ratio of Bcl-2/Bax protein level. ^*^ *p* < 0.05, ^**^ *p* < 0.01, NS: not significant.

We observed that the viability of pericytes markedly (student’s *t* test; *p* < 0.05) decreased as Aβ40 concentration increased (Figure 2a). Moreover, RT-qPCR suggested that the increase of Aβ40 significantly (student’s *t* test; *p* < 0.05) reduced miR-485-5p expression (Figure 2b). Next, as shown in RT-qPCR analysis, miR-485-5p expression was markedly (ANOVA + Tukey’s *post hoc* test; *p* < 0.01) overexpressed by the transfection of miR-485-5p mimics into Aβ40-induced pericytes (Figure 2c). We discovered that the suppressive effect of Aβ40 on pericyte viability was significantly (ANOVA + Tukey’s *post hoc* test; *p* < 0.01) counteracted by overexpressing miR-485-5p (Figure 2d). In addition, flow cytometry analysis suggested that miR-485-5p overexpression markedly (ANOVA + Tukey’s *post hoc* test; *p* < 0.01) reversed Aβ40-induced promotion of pericyte apoptosis (Figure 2e). At last, as shown in Figure 2f, Aβ40-mediated increase in the levels of pro-apoptotic proteins (cleaved caspase-3 and Bax) was significantly (ANOVA + Tukey’s *post hoc* test; *p* < 0.01) reversed by miR-485-5p overexpression. Meanwhile, overexpressing miR-485-5p significantly (ANOVA + Tukey’s *post hoc* test; *p* < 0.01) rescued Aβ40-induced decrease in protein level of Bcl-2, an anti-apoptotic factor.

### 3.3 PACS1 is targeted by miR-485-5p in pericytes

**Figure 3:**
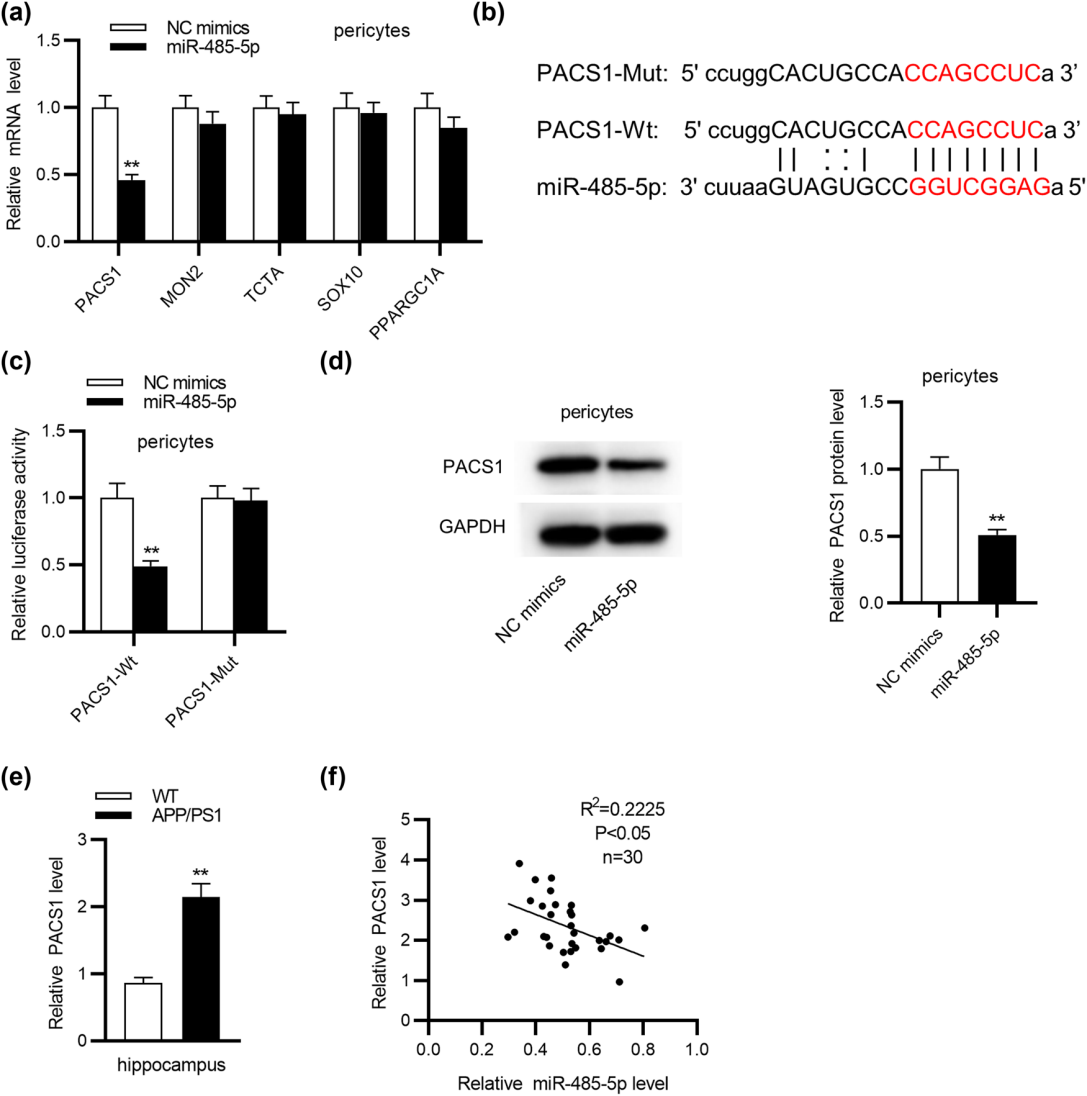
PACS1 is targeted by miR-485-5p in pericytes. (a) The expression levels of PACS1, MON2, TCTA, SOX10, and PPARGC1A in pericytes transfected with miR-485-5p mimics or NC mimics were detected using RT-qPCR analysis. (b) The binding site between PACS1 and miR-485-5p was presented. (c) The luciferase reporter assay was employed to verify the binding between PACS1 and miR-485-5p in pericytes. (d) The protein level of PACS1 in pericytes overexpressing miR-485-5p was assessed using western blot analysis. (e) PACS1 expression in the hippocampus of WT mice or APP/PS1 mice (*n* = 10/group) was assessed using RT-qPCR. (f) The correlation between PACS1 expression and miR-485-5p expression in the hippocampus of APP/PS1 mice was evaluated through Pearson correlation analysis. ^**^ *p* < 0.01.

Based on the bioinformatics tool miRDB, the top five mRNAs (PACS1, MON2, TCTA, SOX10, and PPARGC1A) binding with miR-485-5p were selected for the following study. Among the 5 mRNAs, PACS1 was significantly (student’s *t* test; *p* < 0.01) decreased in pericytes by overexpressing miR-485-5p (Figure 3a). Thus, PACS1 was identified for the following study. The conjectured binding site between miR-485-5p and PACS1 was presented in Figure 3b, and the sequence of PACS1 was mutated. Then, a luciferase reporter assay was adopted to explore the binding between miR-485-5p and PACS1. Compared with the NC group, overexpressed miR-485-5p conspicuously (student’s *t* test; *p* < 0.01) weakened the luciferase activity of the PACS1-Wt vector, while miR-485-5p failed to influence the luciferase activity of PACS1-Mut vector (Figure 3c). Western blot analysis confirmed that overexpressed miR-485-5p markedly (student’s *t* test; *p* < 0.01) decreased the protein level of PACS1 (Figure 3d). In addition, we detected PACS1 expression in the hippocampus of WT mice and APP/PS1 mice and observed that PACS1 expression was significantly (student’s *t* test; *p* < 0.01) higher in the hippocampus of APP/PS1 mice than in that of WT mice (Figure 3e). Furthermore, PACS1 expression was negatively correlated with miR-485-5p expression in the hippocampus of APP/PS1 mice through Pearson correlation analysis (Figure 3f).

### 3.4 PACS1 overexpression countervails the effect of miR-485-5p overexpression on the viability and apoptosis of Aβ40-induced pericytes

**Figure 4:**
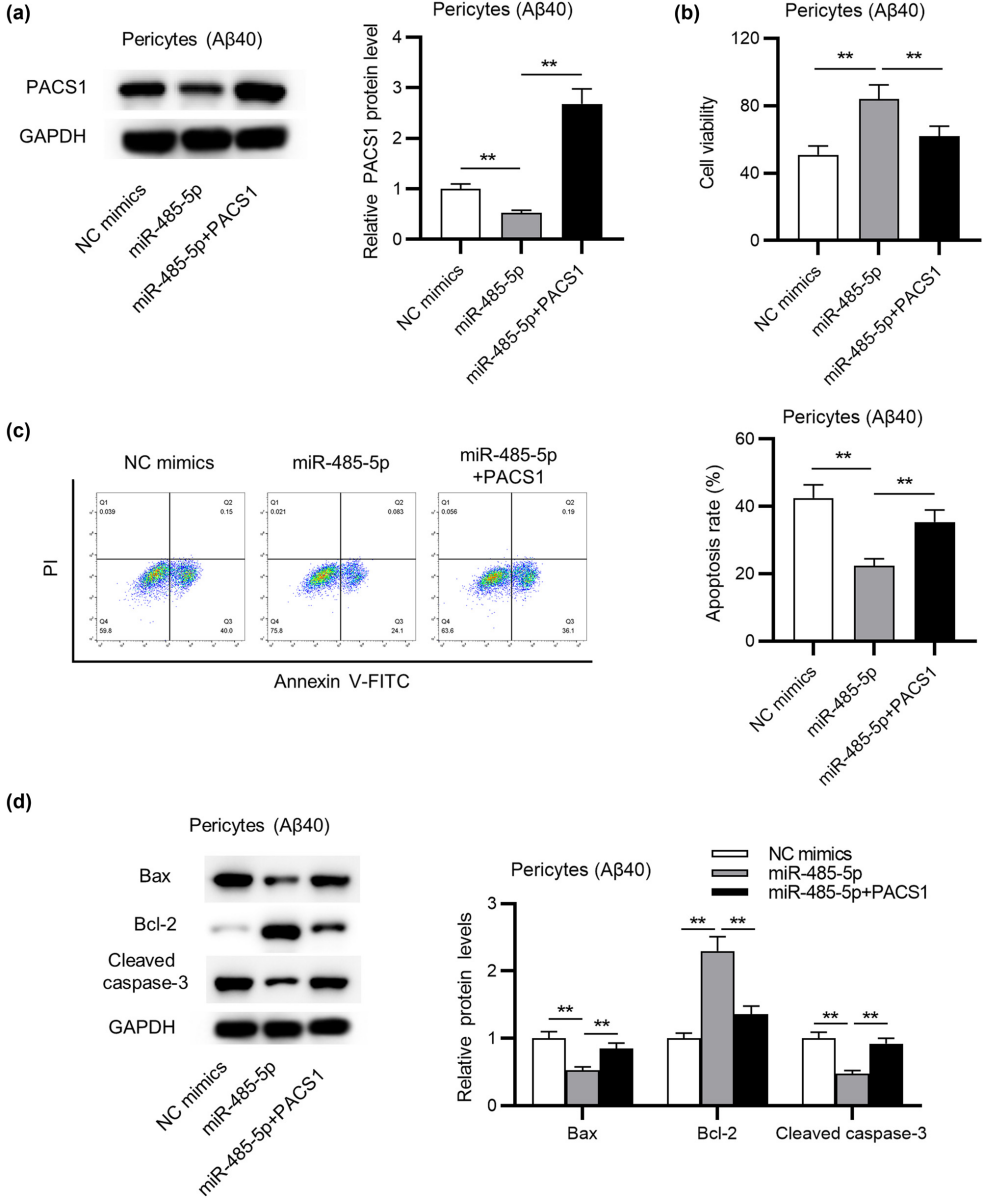
The influence of miR-485-5p overexpression on the viability and apoptosis of Aβ40-induced pericytes can be reversed by PACS1 overexpression. (a) The protein level of PACS1 in Aβ40-induced pericytes overexpressing miR-485-5p or overexpressing both miR-485-5p and PACS1 was detected through western blot analysis. (b) The CCK-8 assay was adopted to evaluate the viability of Aβ40-induced pericytes in the above groups. (c) Flow cytometric analysis was performed to examine the apoptosis of Aβ40-induced pericytes in the above groups. (d) The protein levels of apoptotic markers in pericytes (Aβ40) with the above transfection were assessed by western blot. ^**^ *p* < 0.01.

To further validate the mechanism of miR-485-5p in pericytes, we performed a series of rescue assays. First, western blot analysis revealed that the protein level of PACS1 was successfully (ANOVA + Tukey’s *post hoc* test; *p* < 0.01) increased after transfection of pcDNA3.1/PACS1 into Aβ40-treated pericytes (Figure 4a). Then, CCK-8 assay manifested that the promotive influence of miR-485-5p overexpression on pericyte viability was significantly (ANOVA + Tukey’s *post hoc* test; *p* < 0.01) reversed by overexpressing PACS1 (Figure 4b). Next, flow cytometry analysis reflected that the inhibition of pericyte apoptosis arising from miR-485-5p overexpression was significantly (ANOVA + Tukey’s *post hoc* test; *p* < 0.01) rescued by PACS1 overexpression (Figure 4c). Moreover, miR-485-5p mimics-induced decrease in cleaved caspase-3 and Bax protein levels and augmentation of Bcl-2 level were markedly (ANOVA + Tukey’s *post hoc* test; *p* < 0.01) reversed by PACS1 overexpression, as is shown in western blot analysis (Figure 4d).

## 4 Discussion

AD is the most common form of neurodegenerative dementia [27], with characteristics of memory loss and cognitive deficits such as the impairment of visuospatial skills [28], posing a huge threat to elder people. Though there are no effective treatments for the cure of AD [29,30], the prevention of pericyte apoptosis has been proposed to be efficient in alleviating AD progression [4,31,32].

miRNAs are important regulators involved in AD pathology and development [13]. Previously, miR155 regulates the behavior, neuropathology, and cortical transcriptomics of AD [33]. miR-195 alleviates ApoE4-induced cognitive deficits and lysosomal defects in AD pathogenesis [34]. Moreover, miR-181a was previously reported to protect against pericyte apoptosis for ameliorating cognitive deficits in APP/PS1 mice [25]. Herein, we investigated the effect of miR-485-5p on pericyte apoptosis since miR-485-5p has been confirmed to regulate neuron survival [35]. For example, miR-485-5p was found to control axonal development in hippocampal neurons [36]. miR-485-5p was demonstrated to boost neuron survival after cerebral ischemia/reperfusion [35]. In this study, our results exhibited that miR-485-5p played a suppressive role in AD progression, which might result from its function in inhibiting pericyte apoptosis. Specifically, miR-485-5p expression was found to be downregulated in the hippocampus of APP/PS1 mice. *In vivo* results revealed that overexpressing miR-485-5p improved the learning and memory competence of APP/PS1 mice. Moreover, miR-485-5p overexpression induced the decrease in Aβ42 and Aβ40 concentration and the increase in the number of pericytes. It is known that Aβ deposition is a typical feature of AD pathology [37]. Thus, we concluded that miR-485-5p alleviates the progression of AD *in vivo*. Subsequently, the biological role of miR-485-5p *in vitro* was investigated. Considering that pericyte injury may lead to BBB breakdown, we performed CCK-8 assay to examine pericyte viability. The results suggested that the viability of pericytes decreased in a Aβ40-dependent manner. miR-485-5p overexpression promoted the viability and apoptosis of pericytes treated with Aβ40 *in vitro*.

Mechanistically, miRNAs exert diverse biological functions by regulating gene expression at the posttranscriptional stage [38]. Specifically, miRNAs directly target the 3′-untranslated region (3′-UTR) of specific messenger RNAs (mRNAs) [39]. Previous research verified that miR-485-5p regulates various diseases by targeting specific mRNAs. For example, miR-485-5p plays a tumor-suppressive role in gastric cancer by targeting nucleoside diphosphate-linked moiety X-type motif (NUDT1) [40]. miR-485-5p participated in the mediation of acute myeloid leukemia by targeting interferon-regulatory factor 2 (IRF2) [41]. Accordingly, we hypothesized that miR-485-5p might exert its biological functions on pericytes by targeting a specific mRNA in AD. With bioinformatics tools, we found that PACS1 shares binding site with miR-485-5p. Previously, PACS1 was verified to participate in various biological processes. For example, PACS1 cooperates with HIV-1 to regulate the nuclear export of viral RNA [19]. PACS1 serves as a crucial mediator of BAX/BAK oligomerization and the intrinsic (mitochondrial) pathway to apoptosis [42]. Importantly, PACS1 is verified to regulate the trafficking and processing of the APP, thus affecting AD progression [17,21]. In our study, we identified that PACS1 was targeted by miR-485-5p in the pericytes of APP/PS1 mice. PACS1 expression was upregulated in the hippocampus of APP/PS1 mice. In addition, PACS1 overexpression reversed the promotion of miR-485-5p overexpression on the viability of pericytes (Aβ40) and the suppressive effect of overexpressing miR-485-5p on the apoptosis of pericytes (Aβ40).

In conclusion, miR-485-5p prevents pericytes from apoptosis in AD, which adds a new example showing the significance of pericyte loss in altering AD development. In addition, our data identified that miR-485-5p contributed to the ability of APP/PS1 mice in learning and memory. miR-485-5p may be a crucial mediator of AD development via the regulation of PACS1. The study indicated that miR-485-5p might be a novel target for therapeutic amelioration of AD in the future.
